# Giant Congenital Melanocytic Naevus with Proliferative Nodules Mimicking Congenital Malignant Melanoma: A Case Report and Review of the Literature of Congenital Melanoma

**DOI:** 10.1155/2013/473635

**Published:** 2013-01-16

**Authors:** Massimiliano Scalvenzi, Franco Palmisano, Sara Cacciapuoti, Fiorella Migliaro, Maria Siano, Stefania Staibano, Luigi Tornillo, Claudia Costa

**Affiliations:** ^1^Department of Dermatology, Federico II University, via Pansini 5, 80131 Naples, Italy; ^2^Department of Neonatal Intensive Care, Federico II University, via Pansini 5, 80131 Naples, Italy; ^3^Department of Biomorphological and Functional Science, Federico II University, via Pansini 5, 80131 Naples, Italy; ^4^Institute of Pathology, University of Basel, 4003 Basel, Switzerland

## Abstract

Congenital malignant melanoma (CMM) is a rare condition that is defined as malignant melanoma recognized at birth. CMM may develop in utero in one of three ways: (1) transmission by metastasis through the placenta from a mother with melanoma; (2) primary melanoma arising within a giant congenital melanocytic naevus (GCMN); (3) primary *de novo* cutaneous CMM arising in utero. CMM can be confused clinically and histologically with benign proliferative melanocytic lesions such as giant congenital nevi. We describe the case of a patient presenting a GCMN with proliferative nodules, clinically and dermoscopically resembling a CMM, demonstrating the importance of caution in making a diagnosis of MM and highlighting the possibility that benign lesions as GCMN can mimic a malignant melanoma in this age group.

## 1. Case Report 

**Figure 1:**
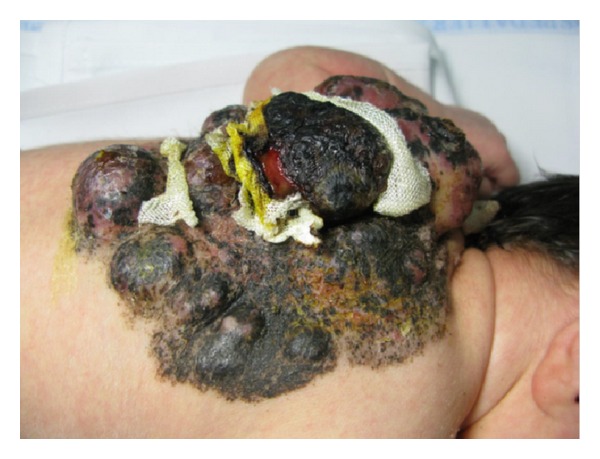
Clinical appearance of a dark, irregular, and raised skin lesion measuring 8 × 11 cm located on the back.

**Figure 2:**
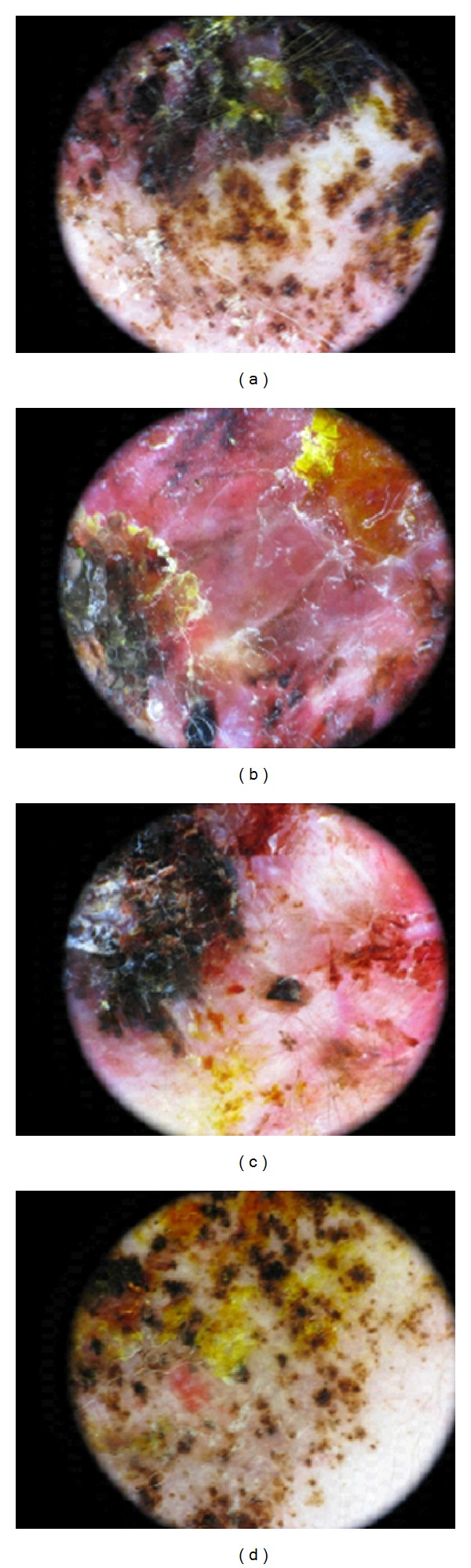
Dermoscopic features. An irregular pigmentation (a–d), atypical pigment network (a–d), irregular dots and globules (a–d), irregular streaks (a–c), and a wide blue-whitish veil (a–c) are clearly visible.

**Figure 3:**
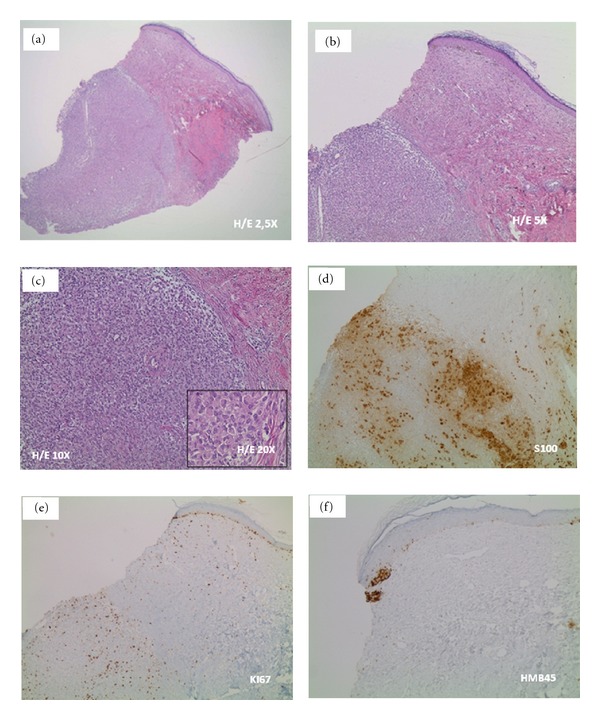
Histopathologic features. We can see (a–c) a dermic component characterized by a solid growth pattern with deep nodules showing a high hypercellularity with no significant atypia. These melanocytes are densely packed and uniform in nature exhibiting a small nucleus; some cells have fine nucleoli. Nuclear pleomorphism is not seen. Immunohistochemical stains. It is visible a strong positivity for S-100 protein (d) and ki67 (e) but a negativity for the HMB-45 (human black melanoma 45) (f). Abbreviations. H/E: hematoxylin and eosin.

**Figure 4:**
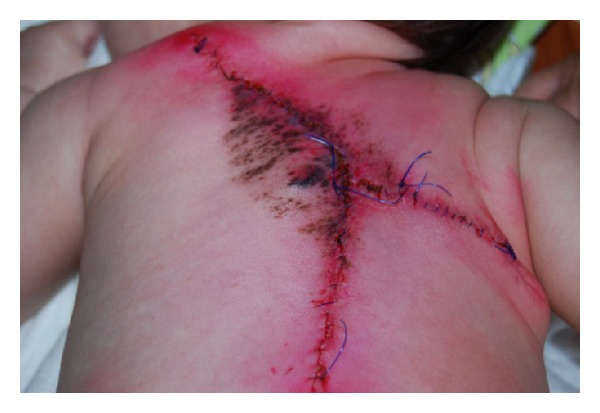
Clinical appearance of the patient after a three-time plastic surgery operation. No skin-grafting or cutaneous expander was needed.

A 7-day-old Italian male child showed at birth a dark, irregular, and raised skin lesion measuring 8 × 11 cm located on the back (Figure 1). He was born full-term by cesarean delivery. The birth weight was 3200 g. He appeared otherwise healthy with no evidence of lymphadenopathy or organomegaly, with an Apgar score of 10. The mother was 30, and she was healthy and received no pharmacological therapies during the pregnancy. There were no maternal suspicious lesions. One month before the delivery, a presumable angiomatous lesion was diagnosed by prenatal ecography. No history of melanoma was known in the family. There were two brothers, without any disease. At the age of 7 days, he was seen at the Department of Dermatology of Federico II of Naples because, according to clinical features of the lesion, there was a very strong suspect of melanoma. A careful dermoscopic examination was performed, which revealed irregular pigmentation, atypical pigment network, irregular dots and globules, irregular streaks, and a wide blue-whitish veil (Figure 2). On the seventh and fourteenth days of life, 4 biopsy specimens of the flat and the raised areas were taken. The specimens were fixed in formalin and sent for histologic analysis. All specimens demonstrated similar histologic features. There was (Figures 3(a), 3(b), and 3(c)) a dermic component characterized by a solid growth pattern with deep melanocytic nodules showing a high hypercellularity with no significant atypia. The melanocytes were densely packed and uniform in nature exhibiting a small nucleus, sometimes with fine nucleoli. Nuclear pleomorphism was not seen. The immunohistochemical stains showed a strong positivity for S-100 protein (Figure 3(d)) and ki67 (Figure 3(e)), while the HMB-45 (human black melanoma 45) staining was negative (Figure 3(f)). A diagnosis of a giant congenital nevus with proliferative dermic nodules was made. There was no histologic evidence of melanoma. A magnetic resonance imaging was performed in order to exclude the presence of brain and spine lesions that can be associated with congenital melanocytic nevus. No leptomeningeal pigmentations or nevi were found in brain and spine. The patient underwent a three-time plastic surgery operation in April, June, and September 2010 that resulted in a complete excision of the lesion (Figure 4). No skin-grafting or cutaneous expander was needed. During a follow-up period of 2 years, this child remained well, with no evidence of malignancy. 

## 2. Discussion and Review 

Congenital melanocytic nevi are present at birth in 1% to 2% of newborns [20], and GCMN, defined as greater than 20 cm in diameter, has a 2% to 42% risk of malignant transformation, with a 6% to 14% lifetime risk of developing melanoma [20, 21]. 

Discrete dermal nodular proliferations commonly referred to as “proliferative nodules” [22], “atypical dermal nodules” [23], “atypical epithelioid tumors in congenital nevi” [24], or “proliferative dermal lesions” [25] can be identified in congenital nevi. 

Both clinical management and histopathologic interpretation of atypical proliferations in congenital melanocytic nevi pose significant challenges to dermatologists and pathologists [20]. 

The true incidence of CMM is difficult to determine due to small number of reported cases and problems associated with diagnosis, and it is likely that some of the cases described as “congenital melanoma” may have been undiagnosed GCMN. 

CMM is extremely rare. From our review of the available literature, twenty cases of CMM have been reported in the English medical literature since 1925 (Table 1). 

Of the 20 children, 12 were males and 4 females (in 4 cases the sex was not reported). Four of the cases were transplacental metastatic melanomas, 9 GCMN-associated melanoma, and 7 *de novo* melanoma. 

Weber et al. in 1930 [15] and Holland in 1949 [16] reported the first CMM arising from maternal malignant melanoma via placental metastasis. Fetal metastasis is extremely rare, and it has been reported that there is about 25% risk of melanoma with placental metastasis spreading from mother to fetus [26–28]. Of course, in such cases the diagnosis is relatively easy. 

Nine neonatal melanomas developed within a GCMN or preexisting nevus; there was evidence of metastasis or local spread in 4 of these patients, 3 of whom subsequently died [29]. 

The other seven cases arose on apparently normal skin, and 3 of these ended in demise of the patient [29]. 

GCMN is a great mimicker of malignant melanoma; clinical indicators such as changes in colour, size, shape, rapid growth rate, nodularity, and even ulceration may occur in this benign lesion. Moreover, melanoma-specific dermoscopic criteria may also be present (Table 2). 

Histologic features recognized as evidence of malignancy like mitotic activity, nuclear pleomorphism, and pagetoid melanocytic proliferation may also be present in a GCMN [29]. Malignant change, however, is exceptional in neonates. 

Previous reports have recognized benign proliferative nodules within GCMNs that behave in a nonaggressive manner [11, 30–32]. Despite their clinically and dermoscopically alarming appearance, in time, these nodules may reduce in size, become softer, and even regress completely, and the histologic features become less worrisome [1, 17, 32]. 

Based on the excellent prognosis of many reported cases, we believe that some previously reported cases of CMM were not malignant lesions. 

We believe that our case represents benign large dermal nodules within GCMN that clinically and dermoscopically resembled a malignant melanoma. 

Dermoscopy is a very useful technique for the analysis of pigmented lesions; it represents a link between clinical and histological views, affording an earlier diagnosis of skin melanoma. 

It also helps in the diagnosis of many other pigmented skin lesions that can mimic melanoma, such as seborrheic keratosis, pigmented basal cell carcinoma, haemangioma, blue naevus, atypical naevus, and benign naevus. 

Cutaneous melanoma can show a multiplicity of characteristics like dermoscopic variation of colours and structures and asymmetry. Dermoscopy facilitates diagnostic suspicion, and can predict the depth of the tumor; for example, melanoma *in situ *and melanoma with dermal invasion exhibit visible differences on close examination. Obviously, its findings have to be confirmed by histopathologic examination [33]. 

Based on our experience and the literature review, we believe that although a lesion can appear alarming, extreme caution is needed in diagnosing a melanoma in an otherwise healthy neonate. 

This paper underlines the importance of a proper diagnosis, for which the histopathological analysis is fundamental; misdiagnosis may lead to anxiety and unnecessary treatment, like chemotherapy and surgical amputations. 

## Figures and Tables

**Table 1:** Review of neonatal malignant melanoma.

Case	Authors	Aetiology	Age at diagnosis	Sex	Location of Lesion	Outcome
1	Coe [1], 1925	*de novo *	Present at birth, diagnosed 8 wk	F	Head	D 10 mo
2	Sweet and Connerty [2], 1941	*de novo *	Present at birth, diagnosed 7 d	M	Buttocks	D 17 d
3	Stromberg [3], 1979	*de novo *	Present at birth, diagnosed 5 mo	No data	Mastoid process	A 18 y
4	Hayes and Green [4], 1984	*de novo *	At birth	M	Disseminated tumor	A 5 y 10 mo
5	Prose et al. [5], 1987	*de novo *	6 wk	F	Abdomen	A 1 y
6	Song et al. [6], 1990	*de novo *	At birth	M	Occiput	D 2 h
7	Asai et al. [7], 2004	*de novo *	2 mo	M	Right thumb	A 3 y
8	Oldhoff and Koudstaal [8], 1968	GCMN	At birth	M	Right thigh	A 10 y
9	Stronmberg [3], 1979	GCMN	At birth	M	Temple	A 6 mo
10	Campbell et al. [9], 1987	GCMN	In utero	M	Mass over spine	D 17 min
11	Naraysingh and Busby [10], 1986	GCMN	At birth	M	Extensive over back containing tumor nodules; multiple satellite lesions	D 6 wk
12	Mancianti et al. [11], 1990	GCMN	8 wk	No data	Right thigh	A 41 mo
13	Mancianti et al. [11], 1990	GCMN	3 wk	No data	Bathing-trunk nevus with nodules	A 18 mo
14	Baader et al. [12], 1992	GCMN	At birth	F	Thoracolumbar and gluteal	A 4 mo
15	Ishii et al. [13], 1991	GCMN	Present at birth, diagnosed 40 d	M	Left thigh	D 18 mo
16	Koyama et al. [14], 1996	GCMN	At birth	F	Scalp with nodules	No data
17	Weber et al. [15] 1930, Holland [16], 1949	Transplacental	8 mo	M	Generalized subcutaneous nodules	D 10 mo
18	Campbell et al. [9], 1987	Transplacental	5 mo	No data	Left upper quadrant	D
19	Dargeon et al. [17], 1950	Transplacental	9 mo	M	Preauricular	D 11 mo
20	Brodsky et al. [18], 1965	Transplacental	11 d	M	Cord blood showed malignant cells; multiple lesions on chest wall	D 7 wk

Abbreviations. GCMN: giant congenital melanocytic nevus; A: alive; D: dead; min: minutes; h: hours; d: days; wk: weeks; mo: months; y: years.

**Table 2:** Seven melanoma-specific dermoscopic criteria [19]. As our case reports, a GCMN may show these features.

Criterion	Definition	Histopathologic correlates
(1) Atypical pigment network	Black, brown, or gray network with irregular meshes and thick lines	Irregular and broadened rete ridges
(2) Blue-whitish veil	Irregular, confluent, gray-blue to whitish-blue diffuse pigmentation	Acanthotic epidermis with focal hypergranulosis above sheets of heavily pigmented melanocytes in the dermis
(3) Atypical vascular pattern	Linear-irregular or dotted vessels not clearly combined with regression structures	Neovascularization
(4) Irregular streaks	Irregular, more or less confluent, linear structures not clearly combined with pigment network lines	Confluent junctional nests of melanocytes
(5) Irregular pigmentation	Black, brown, and/or gray pigmented areas with irregular shape and/or distribution	Hyperpigmentation throughout the epidermis and/or upper dermis
(6) Irregular dots/globules	Black, brown, and/or gray round to oval, variously sized structures irregularly distributed within the lesion	Pigment aggregates within stratum corneum, epidermis, dermoepidermal junction, or papillary dermis
(7) Regression structures	White areas (white scarlike areas) and blue areas (gray-blue areas, peppering, multiple blue-gray dots) may be associated, thus featuring so-called blue-whitish areas virtually indistinguishable from blue-whitish veil	Thickened papillary dermis with fibrosis and/or variable amounts of melanophages
